# Crude Blueberry Phenolic Extracts Improve Gut Barrier Integrity and Exert Anti-Inflammatory and Antimicrobial Activity in an In Vitro Weaning Stress Model

**DOI:** 10.3390/antiox13091044

**Published:** 2024-08-28

**Authors:** Vignesh B. Nathan, Sarah Eckrote, Shiyu Li, Lavanya Reddivari

**Affiliations:** Department of Food Science, Purdue University, 745 Agriculture Mall Drive, West Lafayette, IN 47907, USA; nathanv@purdue.edu (V.B.N.); seckrote@purdue.edu (S.E.); li3291@purdue.edu (S.L.)

**Keywords:** weaning, phenolic compounds, gut health, swine nutrition

## Abstract

Piglet weaning is accompanied by gastrointestinal tract (GIT) dysfunction, resulting in post-weaning diarrhea (PWD). The treatment involves antibiotics due to the susceptibility of the weaned GIT to pathogens. However, antibiotic resistance has shifted attitudes toward a nutraceutical approach by enriching feed with functional compounds. Polyphenols are touted for their antimicrobial activity and ability to improve GIT function. Thus, we investigated the protective effects of crude blueberry phenolic extracts (BPE) in vitro using porcine cells challenged with lipopolysaccharide (LPS) as a weaning model. Cells were pretreated with 1 µg/mL and 2.5 µg/mL BPE for 24 h, followed by 10 µg/mL LPS stimulation for 6 h. Antioxidant status, paracellular permeability, the gene expression of proinflammatory cytokines, and tight junction proteins were measured. The antimicrobial activity of the extract was evaluated against porcine pathogens. The pretreatment of cells with 1 µg/mL BPE preserved catalase (CAT) activity. Reduced paracellular permeability was observed in a dose-dependent manner. The BPE preserved the relative mRNA abundance of tight junctions and reduced inflammatory cytokine expression. Pretreatment with the BPE was able to preserve occludin (OCLN) protein levels. The minimum inhibitory concentration of the BPE against *Enterotoxigenic E. coli* (ETEC) and *Salmonella typhimurium* (ST) was 62.50 µg/mL. These findings indicate that blueberry polyphenols hold potential as feed additives in swine weaning.

## 1. Introduction

Piglets undergo weaning as early as 12 to 45 days post birth [1]. From an economic perspective, early weaning is a crucial step in swine production to improve sow reproductive efficiency [2] and incorporate growth-promoting swine feed. However, during this period, the GIT of these animals begins to change rapidly, with distinct morphological differences. Psychological and dietary stressors are key elements that play a role in regulating GIT development during early life [3]. Thus, weaning is one of the most stressful times for a piglet, and is exacerbated by intestinal dysfunction, bacterial infection, reduced feed intake, and debilitated animal welfare [4]. Among these challenges, impaired GIT function resulting in PWD is the most notable. Theories behind the phenomenon attribute it to infections resulting from common porcine pathogens such as ETEC [5] and ST [6], leading to various systemic symptoms that may cause mortality. Furthermore, GIT immaturity results in a suppressed immune response [7]. Therefore, there is a dire need to strengthen GIT integrity and prevent pathogenic infections during weaning. The GIT consists of a single layer of epithelial cells that are selectively permeable and play an integral role in digestion and adsorption. Paracellular permeability is regulated by three protein complexes identified as desmosomes, adherens junctions, and tight junctions [8]. Among these, tight junctions such as claudin-1 (CLDN-1) and OCLN proteins, are designated as bearing the most workload, with compromised function resulting in the pathogenesis of various intestinal diseases [9]. Therefore, compounds that may upregulate the expression of these proteins have become an area of interest.

Antibiotics at subtherapeutic and therapeutic levels are presently used as the first course of action to mitigate PWD. However, the rise of antibiotic-resistant microorganisms in animal agriculture has shifted attitudes toward the elimination of antibiotic usage where possible. As a result, current trends are focusing on a nutraceutical approach to improve GIT dysfunction associated with weaning stress. Here, swine feed can be enhanced with functional compounds that may decrease villous atrophy and upregulate the expression of tight junction proteins [10].

Whole foods such as fruits (berries, grapes, and apples) and vegetables (carrots, potatoes, and broccoli) contain an array of micronutrients, macronutrients, and bioactive compounds that have been proven to improve intestinal health. These food components may regulate the intestinal barrier via the immunomodulation of inflammatory pathways, promoting healthy gut microbiota, and offering antioxidant properties [11,12,13]. Furthermore, blueberries are rich in anthocyanins, powerful antioxidant polyphenols that may aid in their antimicrobial abilities [14]. Therefore, there has been increased interest in utilizing these compounds as feed additives. Various polyphenols have been readily incorporated into swine feed to reduce oxidative stress in piglets [15,16] while simultaneously serving as growth promoters [17]. Furthermore, previous research has demonstrated that polyphenols can mitigate conditions affecting the porcine gut [18,19], but the data on the mechanisms by which blueberry phenolic compounds provide protection are limited.

In the present study, non-transformed porcine small intestinal epithelial cells (IPEC-J2) isolated from the jejunum of an unsuckled neonatal piglet challenged with LPS were used as a representative model of early weaning stressed piglets. We hypothesize that cells pretreated with the BPE will improve antioxidant status, GIT integrity, and the inflammatory response associated with LPS challenge. In addition, the antimicrobial activity of the BPE on ETEC and ST was evaluated to determine the effectiveness against common PWD-associated pathogens.

## 2. Materials and Methods

### 2.1. Crude Phenolic Extraction of Blueberries

Frozen blueberries were purchased from grocery store and freeze-dried using a freeze dryer (Harvest Right, North Salt Lake, UT, USA). The freeze-dried powder was then extracted for polyphenols using established protocols described by Madiwale and colleagues with slight modifications [20]. In brief, tubes containing five grams of berries were extracted in triplicate using 37.5 mL of (50% Ethanol (EtOH)/50% Water pH = 2.8) solvent. The extracts were vortexed for 20 min, left on ice for 30 min, vortexed for 5 min, and centrifuged at 2500× *g* for 20 min. The supernatant was collected, and 25 mL of acidified water (pH = 2.8) was added to the pellet and heated to 60 °C for 15 min to release bound phenolics. The samples were again centrifuged as previously described and combined with the supernatant. Phenolics were concentrated by combining extracts and utilizing a rotary evaporator set to 40 °C to remove excess solvent. The extracts were stored at −80 °C for future experiments.

### 2.2. Total Monomeric Anthocyanin Content Determination

To quantify the concentration of anthocyanins in the extracts, the BPE was diluted to the appropriate dilution factor using potassium chloride buffer (pH = 1.0) until the absorbance was within the linear range of the spectrophotometer at the λvis-max (approximately 525 nm). Two dilutions were then prepared with potassium chloride buffer (pH = 1.0) and sodium acetate buffer (pH = 4.5), and allowed to equilibrate for 15 min. Extracts were read at 525 nm and 700 nm, and the concentration was calculated using the equation below.

A = (A_525_ − A_700_)_pH 1.0_ − (A_525_ − A_700_)_pH 4.5_

MAC (mg/L) = (A × MW × DF × 1000)/(ε × 1)

where MW is the molecular weight of anthocyanins (cyanidin-3-glucoside, MW = 449.2 g/mol), DF is the dilution factor (DF = 300 for the BPE), and ε is the molar absorptivity (26,900) [17].

### 2.3. Characterization of Blueberry Phenolic Extract

Samples (100 mg) were extracted with 50% methanol containing 0.1% formic acid, vortexed, centrifuged (11,100× *g* for 10 min), and the supernatant was collected. Separations were performed on an Agilent 1290 system (Palo Alto, CA, USA), with a mobile phase flow rate of 0.45 mL/min. The metabolites were assayed using a Waters HSS T3 column (1.8 µm, 2.1 × 100 mm), where the mobile phases A and B were 0.1% formic acid in deionized water and acetonitrile, respectively. Initial conditions were 95:5 A:B, held for 1 min, followed by a linear gradient to 60:40 at 16 min, then 5:95 at 22 min, with a hold until 27 min. Column re-equilibration was performed by returning to 95:5 A:B at 28 min and holding until 32 min. Following chromatographic separation, the column effluent was introduced by positive–electrospray ionization (ESI) into a Q-TOF (Agilent 6546) mass spectrometer. High mass accuracy spectra were collected between 70–1000 *m*/*z*. To assist with compound identification, samples were assayed in data-dependent MS/MS acquisition mode. Peak finding was performed using the open-source MS-DIAL bioinformatics software version 5.3.240719. Masses were annotated by querying the metabolite library MoNA, with a mass tolerance of 0.005 Da.

### 2.4. Cell Culture and Treatments

IPEC-J2 cells from the DSMZ German Collection of Microorganisms and Cell Cultures ACC 701 were a generous gift from Dr. Arun K. Bhunia (Purdue University, West Lafayette, IN, USA). The IPEC-J2 cells were maintained in Roswell Park Memorial Institute medium (RPMI-1640) (Thermo Fisher Scientific, Waltham, MA, USA), with 10% fetal bovine serum (FBS) (Atlanta Biologicals, Flowery Branch, GA, USA) and five ng/mL epidermal growth factor (EGF) (Corning Inc., Corning, NY, USA) in a humidified cell culture incubator set at 37 °C with 5% CO_2_. Cells with a passage lower than 25 were used for this study. Unless otherwise stated, cells were seeded on six-well plates (Techno Plastic Products, Trasadingen, Switzerland) at a density of 0.3 × 10^6^ cells/mL and grown in RPMI-1640 with FBS and EGF to approximately 80% confluency. Monolayers were then pretreated with 1 µg/mL and 2.5 µg/mL BPE resuspended in serum-free and EGF-free medium for 24 h then challenged with 10 µg/mL LPS from *Escherichia coli* O11:B4 (Millipore Sigma, St. Louis, MO, USA) for 6 h.

### 2.5. Cytotoxicity Assay

To determine if the BPE has any cytotoxic effects, the cells were seeded on 24-well plates (Techno Plastic Products, Trasadingen Switzerland) and treated in a dose-dependent manner with the BPE. Supernatants were collected from each well and measured for lactate dehydrogenase (LDH) activity using an LDH Cytotoxicity Assay Kit from Cayman Chemical Company (Radnor, PA, USA). A 1% Triton X-100, Surfact-Amps Detergent Solution (Thermo Fisher Scientific, Waltham, MA, USA), in cell culture media served as a positive control.

### 2.6. Catalase Activity Assay

LPS is known to increase oxidative stress. Thus, to determine the antioxidant status of cells, a catalase (CAT) activity kit was purchased from Invitrogen (Waltham, MA, USA). After the scheduled termination of the experiment, cells were harvested utilizing the assay buffer and treated with hydrogen peroxide. The absorbance (560 nm) was measured according to manufacturer protocols.

### 2.7. Paracellular Permeability Assay

Epithelial integrity is crucial as the first line of defense against foreign agents. Therefore, to measure paracellular permeability, cells were seeded onto 0.9 cm^2^ transwell inserts with a pore size of 3.0 µm (Corning Inc., Corning, NY, USA) on 12-well plates (Techno Plastic Products, Trasadingen, Switzerland) at a density of 1 × 10^5^ cells/mL. Cell culture media was replaced every three days to induce differentiation. Trans-epithelial electrical resistance (TEER) was measured using an EVOM3 epithelial volt-ohm meter (World Precision Instruments, Sarasota, FL, USA) prior to the start of the experiment. Transwells that had a TEER value of 800 Ohm × cm^2^ (Ωcm^2^) or greater only were used. On day 21 post differentiation, cell monolayers were challenged with 10 µg/mL LPS on the apical side for 6 h. Immediately afterward, 1 mg/mL 4-kDa fluorescein isothiocyanate-dextran (FITC) (Millipore Sigma, St. Louis, MO, USA) dissolved in RPMI-1640 was added to the apical side, and the amount of fluorescence was measured on the basolateral side with excitation and emission wavelengths of 485 nm and 528 nm, respectively, using a BioTek Cytation 1 Imaging Reader (BioTek, Winooski, VT, USA).

### 2.8. RNA Extraction and Real-Time Polymerase Chain Reaction (RT-PCR) Analysis

The gene expressions of tight junction proteins and proinflammatory cytokines were measured using an Applied Biosystem QuantStudio 3 RT-PCR System (Thermo Fisher Scientific, Waltham, MA, USA). The total RNA was extracted after experiment termination with a RNeasy Micro Kit (Quiagen, Hilden, Germany). The purity was confirmed using a nucleic acid quantification plate in a BioTek Cytation 1 Imaging Reader (BioTek, Winooski, VT, USA), with absorbances read at 280 nm and 260 nm wavelengths. The RNA was then synthesized with a High-Capacity cDNA Reverse Transcription Kit purchased from Thermo Fisher Scientific (Waltham, MA, USA) according to the manufacturer’s protocols. RT-PCR was performed using primers described in Table 1, taken from Yan and colleagues [21] using SYBR green (Thermo Fisher Scientific, Waltham, MA, USA). The RT-PCR protocol consisted of a denaturing stage at 95 °C, followed by annealing at 56 °C and extension at 72 °C, for a total of 50 cycles. The mRNA expression was normalized to β-actin. Primers were purchased through Integrated DNA Technologies (Coralville, IA, USA), and primer efficiency, primer dimerization, and melt curve analysis were performed before running extracted samples. The gene expression was calculated using the formula 2^−ΔΔCt^ method and the relative quantity was reported.

### 2.9. Western Blot Analyses of Tight Junction Proteins

Phenolic compounds can upregulate the expression of tight junction proteins in an inflammatory model [22]. Thus, the expression of OCLN was determined through immunoblotting. Treated cells were washed with phosphate-buffered saline (PBS) and lysed using ice cold RIPA lysis and extraction buffer (Thermo Fisher Scientific, Waltham, MA, USA) containing halt protease and phosphatase inhibitors (Thermo Fisher Scientific, Waltham, MA, USA). The total protein concentration of the cell lysates was quantified using a Pierce BCA protein assay kit (Thermo Fisher Scientific, Waltham, MA, USA) according to the manufacturer’s protocols, separated using SDS-PAGE gels (4–12% polyacrylamide), and transferred to the polyvinylidene difluoride membrane (Millipore Sigma, St. Louis, MO, USA). The membranes were blocked using non-fat dry milk in 0.1 M Tris-buffered saline with 0.1% Tween 20 for 1 h. The membranes were then incubated with mouse anti-Pig OCLN (Cat# 50-173-6807, 1:500 dilution, Thermo Fisher Scientific, Waltham, MA, USA) and mouse anti-mouse β-actin (Cat# AM1021B, 1:500 dilution, Abcepta, San Diego, CA, USA). A secondary goat anti-mouse IgG (Cat# PI31430, 1:1000 dilution, Thermo Fisher) was used for detection. Various antibodies were probed using the same membrane by using Restore Western Blot Stripping Buffer (Thermo Fisher Scientific, Waltham, MA, USA) according to the manufacturer’s instructions. The band intensity was measured using Quantity One software version 4.6.8 (Bio-Rad, Hercules, CA, USA).

### 2.10. Minimum Inhibitory Concentration Assay

To determine if the BPE had any antimicrobial properties, a minimum inhibitory concentration assay was performed. Bacterial strains were acquired from American Type Culture Collection (ATCC, Manassas, VA, USA) ETEC ATCC 35401 and ST ATCC 14028. Strains were grown in tryptic soy broth (Fisher Scientific, Hampton, NH, USA) with yeast extract overnight in a 37 °C incubator. The bacterial cells were washed with sterile PBS and reconstituted in Mueller Hinton broth (Fisher Scientific, Hampton, NH, USA). A 200 µL of bacterial suspension was loaded onto a 96-well plate at 5 × 10^5^ CFU/mL. The BPE was serially diluted in the wells, ranging from 500 µg/mL to 0.97 µg/mL, and 50 µL was added. Wells not containing the BPE and wells containing the serially diluted BPE with no bacteria were used as blanks to control the intrinsic color of the extract. The plate was incubated for 24 h in a 37 °C incubator and absorbance measurements were taken at 600 nm to determine bacterial growth.

### 2.11. Statistical Analysis

Experimental data were analyzed using GraphPad Prism version 9.5.1 (La Jolla, CA, USA) software. The data for experiments are presented as the mean ± standard error (SE) of three independent experiments, where n = 3 refers to three biological plate replicates with three technical replicates per plate for cell culture experiments. The well averages from each plate were then analyzed for statistical significance using one-way analysis of variance (ANOVA), followed by Tukey’s multiple comparison test. All data were normally distributed following an analysis of the resultant QQ plot. Values of *p* < 0.05 were considered statistically significant.

## 3. Results

### 3.1. Determination of BPE Phenolic Compounds

Liquid chromatography of the BPE identified a variety of different metabolites including flavonoids, flavanols, and other phytochemicals (Table 2). Of importance, the extract consisted of anthocyanidins delphinidin, petunidin, cyanidin, and peonidin and their glycosides, which exert strong biological activities pertaining to inflammation and antioxidant status [19]. Moreover, other pertinent metabolites present in the extract included hydroxycinnamic acids and flavonoids both of which have positive effects on improving tight junction protein expression in a weaning piglet model [23,24].

### 3.2. BPE Is Non-Cytotoxic to IPEC-J2 Cells at Low Concentrations

**Figure 1:**
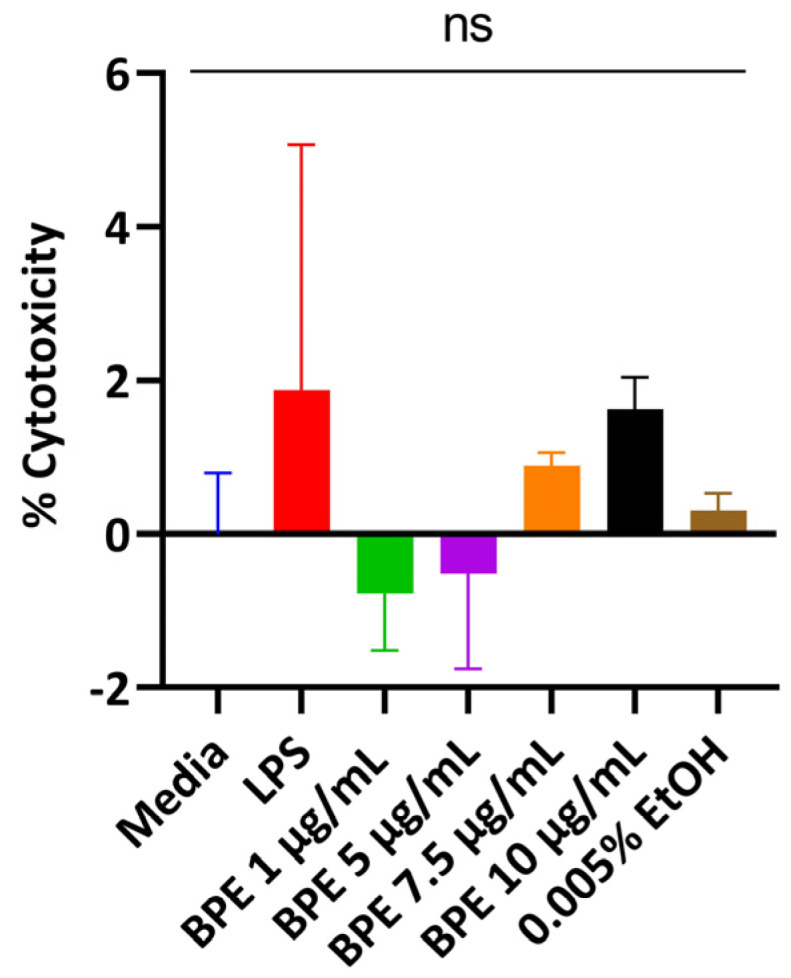
Dose-dependent response of the BPE and treatments on cell viability measured through LDH activity. The data are the average of three independent trials, n = 3.

Cells release LDH when the plasma membrane is damaged, which is indicative of cellular stress. Thus, cells were treated with the BPE in a dose-dependent manner and supernatants were assayed. The BPE did not elicit a strong LDH response with rising concentrations compared to the untreated media control (Figure 1). A concentration of 0.005% EtOH was used to ensure residual solvent from extraction did not injure the cells. However, the monolayer began peeling at 5 µg/mL BPE and higher resulting in low LDH activity. 1 µg/mL and 2.5 µg/mL were used for subsequent experiments.

### 3.3. BPE Pretreatment Conserves Antioxidant Status after LPS Challenge at Low Concentrations

**Figure 2:**
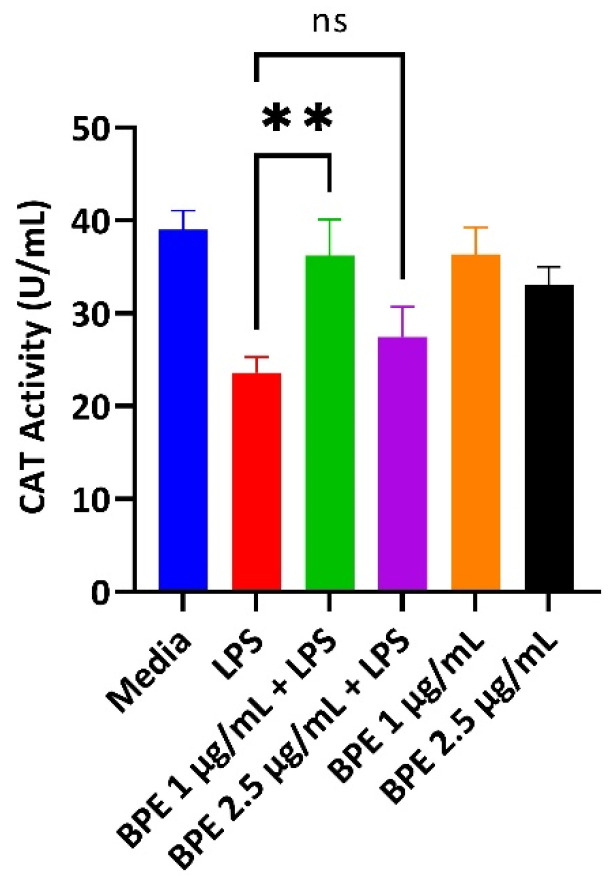
The BPE restores LPS-induced reduction of CAT activity. 1 µg/mL BPE pretreatment preserves the antioxidant status of IPEC-J2 cells. Data are the average of three independent trials, n = 3. **, *p* = 0.0012.

LPS induces the production of reactive oxygen species (ROS). Thus, antioxidant enzymes that can scavenge these free radicals are paramount to cellular survival. A CAT assay was performed with pretreatment of the BPE followed by exposure to LPS to assess the ability to combat ROS revealing preservation of CAT activity. Here, CAT activity assay showed significant conservation of the enzyme after pretreatment with the BPE at 1 µg/mL (Figure 2). Extracts alone did not significantly upregulate CAT activity.

### 3.4. BPE Pretreatment Reduces Paracellular Permeability after LPS Challenge

**Figure 3:**
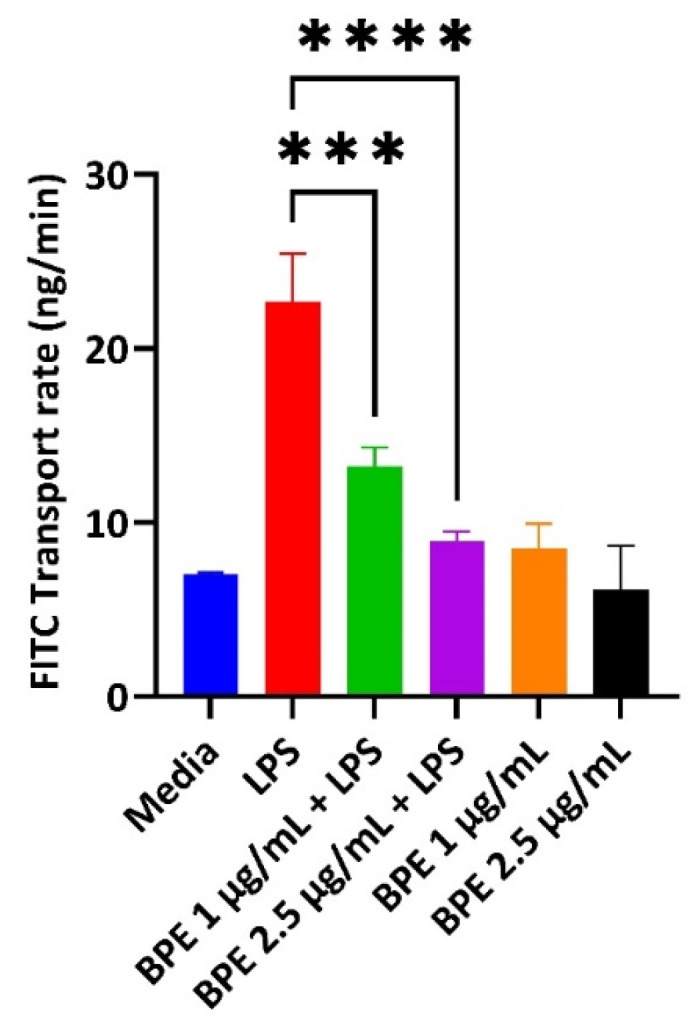
The BPE impact on IPEC-J2 paracellular permeability. Pretreatment with the BPE preserves the epithelial barrier integrity of IPEC-J2 cells. Data are the average of three independent trials, n = 3. *** *p* = 0.0002 and **** *p* < 0.0001.

LPS can destroy the intestinal barrier leading to an increase in paracellular permeability. Deterioration of the epithelial cells in turn results in the pathogenesis of inflammatory conditions and decreased expression of tight junction proteins. Therefore, BPE efficacy in restoring GIT integrity was measured using a FITC permeability assay (Figure 3). Pretreatment with the BPE alone did not change the diffusion of FITC compared to the control. However, BPE at 1 µg/mL and 2.5 µg/mL dose-dependently reduced the increased permeability associated with LPS challenge.

### 3.5. BPE Pretreatment Decreases Expression of Inflammatory Markers and Upregulates Expression of Tight Junction Proteins

**Figure 4:**
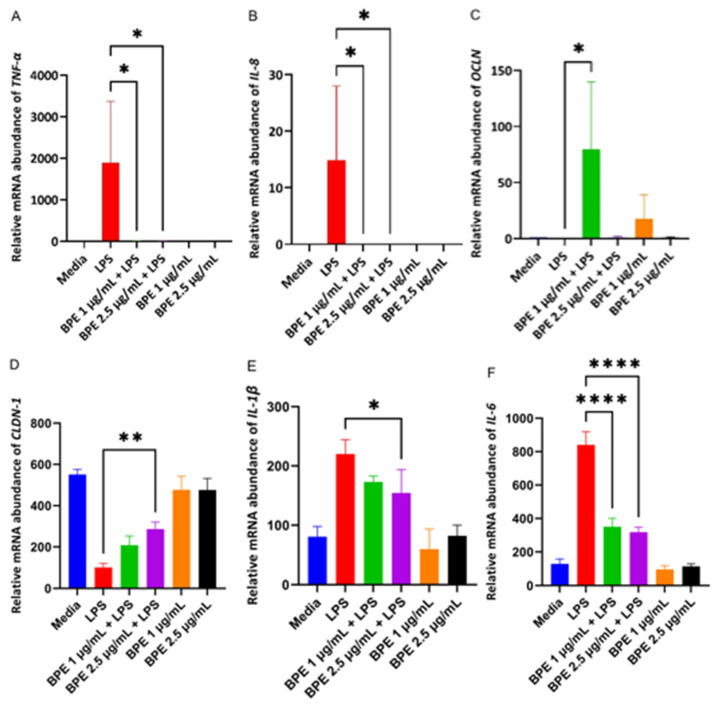
The BPE pretreatment affects gene expression of inflammatory cytokines and tight junction proteins. The relative gene expressions of proinflammatory cytokines and tight junction proteins TNF-α (**A**) *, *p* = 0.0233 IL-8 (**B**) *, *p* = 0.0460, OCLN (**C**) *, *p* = 0.0267, CLDN-1 (**D**) **, *p* = 0.0045, IL-1β (**E**) *, *p* = 0.0045, IL-6 (**F**) ****, *p* < 0.0001. Data are the average of three independent trials, n = 3.

Cells challenged with LPS are susceptible to inflammation which is mediated by pro-inflammatory cytokines such as TNF-α, and interleukins (IL) IL-8, IL-6, and IL-1β. The inflammatory markers can also negatively impact intestinal epithelial tight junction permeability. Thus, RT-PCR was used to evaluate the effect the BPE had on relative mRNA abundance of proinflammatory cytokines and tight junction proteins (Figure 4). As expected, LPS increased the relative abundance of TNF-α, IL-8, IL-6, and IL-1β. Only 2.5 µg/mL BPE was sufficient to reduce IL-1β with both concentrations suppressing expression of other inflammatory cytokines. LPS decreased relative expression of tight junction proteins, however, pretreatment with 1 µg/mL BPE was able to increase OCLN expression whereas 2.5 µg/mL BPE was effective in CLDN-1 expression.

### 3.6. Pretreatment with BPE Is Able to Preserve the Expression of Tight Junction Protein OCLN

**Figure 5:**
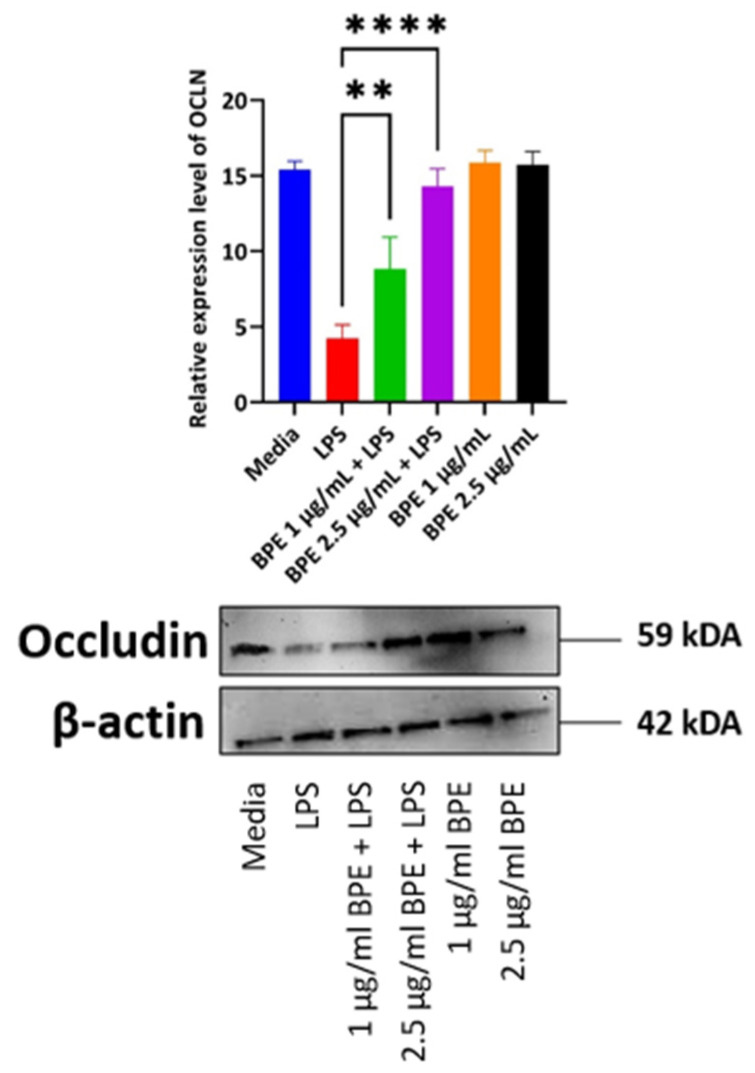
The BPE preservation of OCLN protein. Protein levels were normalized to housekeeping protein β-actin. Data is the average of three independent trials, n = 3. ** *p* = 0.0045 and **** *p* < 0.0001.

LPS disrupts tight junction protein regulation and thus increases paracellular permeability to foreign agents as noted by decreased expression in the LPS only treated group (Figure 5). This phenomenon is a hallmark of a variety of intestinal disorders and thus compounds, which can preserve tight junction protein expression are vital. Here, the BPE was able to preserve tight junction protein OCLN expression dose-dependently with the higher concentration having a more pronounced effect.

### 3.7. BPE Exhibits Antimicrobial Activity against Common PWD Pathogens

**Figure 6:**
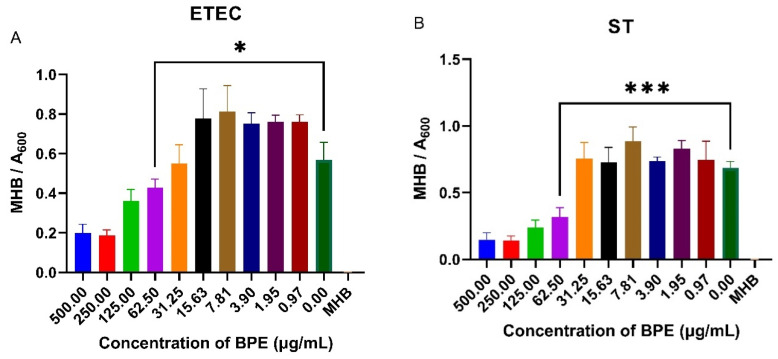
Minimum inhibitory concentration of the BPE against ETEC (**A**) and ST (**B**) in Mueller Hinton broth. Data are the average of three independent trials, n = 3. * *p* = 0.0346 and *** *p* = 0.0008.

PWD is commonly associated with infection of porcine pathogens such as ETEC and ST. Knowing that anthocyanin-rich crude blueberry extracts have shown to reduce pathogenic bacterial growth, the antimicrobial possibility of the BPE was investigated by performing a minimum inhibitory concentration assay on ETEC and ST. The BPE reduced bacterial growth at higher concentrations, with a slight increase in growth at smaller doses (Figure 6). The minimum inhibitory concentration of the BPE was found to be 62.50 µg/mL for both pathogens, with increased suppression of ST suggesting the extract has potential antimicrobial activity.

## 4. Discussion

A diminished GIT barrier function is associated with the pathogenesis of a variety of intestinal disorders. Early weaning-stressed piglets are no different and experience impaired GIT integrity due to the dietary and psychological stressors during weaning. As a result, pathogenic bacteria, antigens, and other foreign agents readily translocate across the GIT [25], activating an immune response that may be damaging to piglets. PWD remains a major concern for weaning piglets, with an estimated 20–30% of cases proving fatal [26]. Therefore, the search for an antibiotic alternative to mitigate the onset of the disease has been crucial in the animal agriculture community. The nutraceutical industry has seen a rapid expansion in functional foods that can improve gut health. As such, functional food ingredients have been evaluated as potential feed additives. Blueberries are fruits that are high in anthocyanins and other polyphenolic compounds (Table 2) which have merit for their antioxidant and anti-inflammatory properties [27,28].

In the present study, IPEC-J2 cells challenged with LPS were used as an experimental model to determine the effects of crude blueberry phenolic extracts at nontoxic working concentrations (Figure 1) on reducing inflammation, restoring intestinal barrier function, and preserving antioxidant status. These cells have been used extensively in studies due to their close similarity to human physiology, making them an ideal in vitro model for epithelial functionality [29].

The characterization of the BPE revealed several classes of polyphenols, including anthocyanidins and delphinidin being the most abundant. Delphinidin is widely regarded for its array of anti-inflammatory capabilities and ability to shift gut microbiota. Metabolization of the compound in the colon is achieved through beneficial *Lactobacillus* species, allowing for increased bioavailability [30]. This further highlights the potential applicability of the extract to select healthy intestinal bacteria in weaning piglets, whose gut microbiome are severely altered during the weaning period. In addition, the crude extract contained varying amounts of anthocyanins, such as cyanidin-3-O-alpha-arabinopyranoside. This is notable due to the anthocyanins being more water soluble and thus more sensitive to degradation due to pH and temperature. While less stable than their anthocyanidin counterparts, anthocyanins still play a leading role in remediating GIT dysfunction [31]. Furthermore, other phenolic compounds such as chlorogenic acid were present. The compound has various biological activities but is widely regarded as a potent antioxidant through direct scavenging of free radicals [32]. Chen and colleagues demonstrated that supplementation with chlorogenic acid reduced oxidative stress in diquat challenged pigs as well as in upregulated tight junction proteins, enhancing barrier integrity [33]. Thus, the beneficial effects of the BPE on LPS challenged cells may be attributed to the combination of various metabolites in different quantities rather than one sole contributor.

Here, the BPE at 1 µg/mL was able to attenuate the reduction of antioxidant enzymes from LPS challenged cells (Figure 2). Weaning pigs are under immense oxidative stress, which can injure cell membranes by disrupting tight junctions [34]. Therefore, compounds that can digest ROS are one mechanism for improving GIT integrity. Interestingly, 2.5 µg/mL BPE did not have a significant effect on CAT activity. One explanation is there was not enough ROS generation with pretreatment and thus no need for the enzyme to be activated.

Intestinal permeability is a key symptom associated with PWD as the piglets have an immature GIT that is further inundated with stressors. The “leaky” gut is then more susceptible to the translocation of dietary antigens or pathogenic bacteria [35]. Therefore, strengthening gut barrier integrity is an essential first line of defense. Here we have shown that the BPE extracts were able to preserve paracellular permeability, as assessed by the FITC in a dose dependent manner (Figure 3). One potential mechanism of action is through the upregulation of tight junction proteins, such as OCLN. Previous work has utilized sour cherry anthocyanin extracts on LPS-challenged human colon epithelial cells and showed the compounds could reduce the associated monolayer permeability, which confirmed this mechanism [36]. Based on our results, 1 µg/mL BPE was able to significantly upregulate the relative mRNA abundance of OCLN and CLDN-1 (Figure 4C,D), while both concentrations were able to preserve OCLN protein expression dose-dependently (Figure 5).

Piglets also secrete a variety of proinflammatory cytokines in a transient manner during weaning that is associated with intestinal inflammation [37]. Recently, nutritional interventions during this phase have been implemented to reduce these markers. In our study, the BPE at both concentrations reduced TNF-α, IL-8, and IL-6 mRNA abundance (Figure 4A,B,F). However, only the higher dose of 2.5 µg/mL BPE was enough to significantly reduce IL-1β (Figure 4D). The findings suggest that the extracts do possess an anti-inflammatory role. Taken together, the preservation of tight junction protein expression and reduction of the proinflammatory cytokines can contribute to the protection of GIT health during the weaning period, with these markers heavily impacting the paracellular permeability and damage to GIT morphology of early weaned piglets [38].

PWD is commonly associated with infections of ETEC and ST, and if overall pathogenic bacterial infections can be controlled, symptoms of PWD could be lessened. Knowing that anthocyanin-rich crude blueberry extracts have shown to reduce and, at times, inhibit pathogenic bacterial growth, the antimicrobial possibility of the BPE was assessed. The minimum inhibitory concentration of the BPE was found to be 62.50 µg/mL for both ETEC and ST (Figure 6). This shows that the extract has the potential to inhibit common PWD pathogens. Interestingly, the BPE increased bacterial growth at lower doses against the zero dose, which may be attributed to the bacteria utilizing the inherent sugars present as an energy source. A complementary study using sucrose to control sugar levels indicated no significant increase in bacterial growth at lower concentrations because of polyphenols. (Data available upon request).

## 5. Conclusions

With the limitations placed on antibiotic usage, farmers and animal health companies alike are searching for alternatives to help piglets with PWD. In this study, polyphenolic extracts from blueberries were able to improve the antioxidant status, decrease paracellular permeability and inflammation in vitro while also preserving tight junction protein expression. Furthermore, high doses of the extract exhibited antimicrobial activity. These findings suggest that functional feed rich in polyphenols could be used as a possible nutritional intervention in early weaning-stressed piglets.

## Figures and Tables

**Table 1:** Primer sequences used for quantitative real-time PCR.

Genes	Genes Accession Number	Primer Sequences
TNF-α	P23563	F: 5′-ATGGATGGGTGGATGAGAAA-3′ R: 5′-TGGAAACTGTTGGGGAGAAG-3′
IL-8	P26894	F: 5′-CACCTGTCTGTCCACGTTGT-3′ R: 5′-AGAGGTCTGCCTGGACCCCA-3′
OCLN	A0A287AL69	F: 5′-GAGAGAGTGGACAGCCCCAT-3′ R: 5′-TGCTGCTGTAATGAGGCTGC-3′
IL-1β	P26889	F: 5′-CCAAAGAGGGACATGGAGAA-3′ R: 5′-GGGCTTTTGTTCTGCTTGAG-3′
IL-6	P26893	F: 5′-TCTGGGTTCAATCAGGAGACCTGC-3′ R: 5′-TGCACGGCCTCGACATTTCCC-3′
CLDN-1	A0A287A1F1	F: 5′-TTTCCTCAATACAGGAGGGAAGC-3′ R: 5′-CCCTCTCCCCACATTCGAG-3′
β-actin	Q80X90	F: 5′ AGCCATGTACGTAGCCATCC-3′ R: 5′-CTCTCAGCTGTGGTGGTGAA-3′

**Table 2:** High-performance liquid chromatography of the metabolites present in the BPE extract. Data are presented as the area under the curve (±) SE of three individual replicates.

Metabolite	Area Under Curve (±) SE
*Hydroxycinnamic acids*	
3-Hydroxycinnamic acid	15,033 ± 188
Caffeic acid	91,956 ± 215
*Flavonoid-3-O-glycosides*	
Isoquercitrin	4663 ± 806
Quercetin-3-O-xyloside	151,812 ± 3697
Isorhamnetin-3-O-beta-D-Glucoside	328,457 ± 4587
Cyanidin-3-O-alpha-arabinopyranoside *	1,558,051 ± 3961
Quercetin-3-O-xyloside	7692 ± 295
5,7-dihydroxy-2-[4-hydroxy-3-[(2S,3R,4S,5R)-3,4,5-trihydroxyoxan-2-yl]oxyphenyl]-3-methoxychromen-4-one	119,690 ± 1817
Cyanidin-3-O-alpha-arabinopyranoside *	5358 ± 278
Peonidin-3-O-alpha-arabinopyranoside *	70,535 ± 846
Quercetin-3-O-glucosyl-6′′-acetate	80,661 ± 993
Peonidin-3-O-alpha-arabinoside *	10,584 ± 465
Myricetin-3-Xyloside	9074 ± 485
Isoquercetin	123,995 ± 2503
Quercetin-3-Arabinoside	29,406 ± 988
Quercetin 3-O-malonylglucoside	8552 ± 223
Kaempferol-3-glucoside	5162 ± 49
5,7-dihydroxy-2-(4-hydroxy-3-methoxyphenyl)-3-[3,4,5-trihydroxy-6-[[(2R,3R,4R,5R,6S)-3,4,5-trihydroxy-6-methyloxan-2-yl]oxymethyl]oxan-2-yl]oxychromen-4-one	4754 ± 266
Quercetin-3-O-alpha-L-rhamnopyranoside	32,385 ± 1379
Isorhamnetin-3-O-beta-D-Glucoside	6642 ± 94
Quercetin-3-O-glucosyl-6′′-acetate	5422 ± 157
Quercetin-3-O-glucosyl-6′′-acetate	9679 ± 443
2-(3,4-dihydroxyphenyl)-5,8-dihydroxy-7-methoxy-3-[(2S,3R,4R,5R,6S)-3,4,5-trihydroxy-6-methyloxan-2-yl]oxychromen-4-one	6220 ± 303
*Flavonoid-3-O-glucuronides*	
Quercetin 3-O-glucuronide	66,122 ± 1386
Kaempferol 3-glucuronide	6642 ± 94
*Flavonoid-7-O-glycosides*	
Nepetin-7-glucoside	3270 ± 430
Plantaginin	240,153 ± 8929
NCGC00380911-01!2-(3,4-dihydroxyphenyl)-3,5-dihydroxy-8-methoxy-7-[(2S,3R,4S,5S,6R)-3,4,5-trihydroxy-6-(hydroxymethyl)oxan-2-yl]oxychromen-4-one	18,211 ± 321
*Anthocyanidin-3-O-glycosides*	
Delphinidin 3-glucoside *	3557 ± 588
Petunidin-3-O-beta-glucoside *	2141 ± 393
Delphinidin-3-O-beta-glucopyranoside *	370,983 ± 6428
Peonidin-3-o-beta-d-glucopyranoside *	148,567 ± 3296
*Flavonols*	
Quercetin	31,625 ± 245
Kaempferol	22,206 ± 854
Isorhamnetin	25,146 ± 999
Myricetin	9599 ± 225
Isorhamnetin	6573 ± 357
Limocitrin	11,597 ± 481
*Flavones*	
Luteolin	18,168 ± 243
*Flavanones*	
2-(3,4-dihydroxyphenyl)-3,5,7-trihydroxy-4H-chromen-4-one	5354 ± 223
*Catechins*	
Epicatechin	20,234 ± 562
*Epigallocatechins*	
Epigallocatechin	4253 ± 217
*Quinic acids and derivatives*	
Chlorogenic acid	3354 ± 254
*Biflavonoids and polyflavonoids*	
Procyanidin B1	25,277 ± 688
*Phenolic glycosides*	
(E)-3-[4-[(2S,3R,4S,5S,6R)-3,4,5-trihydroxy-6-(hydroxymethyl)oxan-2-yl]oxyphenyl]prop-2-enoic acid	5894 ± 395
*6-O-methylated flavonoids*	
4′,5,7-trihydroxy-3,6-dimethoxyflavone	14,776 ± 448

* Samples were run without the addition of hydrogen.

## Data Availability

All data available upon request.
